# Trichobezoar gastrique - à propos de deux cas

**Published:** 2010-09-19

**Authors:** Hassani Karim Ibn Majdoub, Bouhaddouti Hicham El, Benamar Youssef, Mazaz Khalid, Taleb Khalid Ait

**Affiliations:** 1 Service de chirurgie B, CHU Hassan II, Fès, Maroc; 2 Service de chirurgie A, CHU Hassan II, Fès, Maroc,; 3 Service de Chirurgie, Hôpital el Ghassani, Fès, Maroc

**Keywords:** Trichobezoa, Trichophagie, Gastrique, Exérèse chirurgicale, Maroc

## Abstract

**Abstract:**

Le trichobezoar gastrique est une affection rare (un peu plus d’une dizaine de cas dans la littérature), qui affecte essentiellement des jeunes filles perturbées par des désordres psychologiques. Les auteurs rapportent deux cas de jeunes filles, hospitalisées pour volumineuse masse épigastrique. La fibroscopie gastrique a posé le diagnostic de trichobezoar. Une exérèse chirurgicale a été réalisée à travers une gastrotomie, sans complications. Un suivi psychiatrique des deux patientes a été recommandé. Le trichobezoar gastrique désigne l'accumulation inhabituelle de cheveux au niveau de l’estomac. Son diagnostic est facile en présence d’un contexte de trichophagie évocateur. La fibroscopie œsogastroduodénale est l’examen de référence permettant la visualisation du trichobezoar dont le traitement est essentiellement chirurgical.

## Introduction

Le Trichobezoar gastrique est une affection rare désignant la présence inhabituelle de cheveux, sous forme de masse solide, au niveau de l'estomac. Cliniquement asymptomatique, son diagnostic repose essentiellement sur la fibroscopie et son traitement est essentiellement chirurgical. A travers deux cas de Trichobezoar gastrique, les auteurs discutent, en dehors d’un contexte évocateur, les difficultés diagnostic et thérapeutiques posées par cette pathologie. Les auteurs déclarent avoir reçu le consentement écrit des patientes pour reporter ces cas.

## Patient et observation clinique

**Observation nº1**

Il s'agit d'une fillette de 9 ans, ayant comme antécédents chargés, tous d’abord une trichophagie depuis l'âge de 5ans, une anémie associée à un ictère à l'âge de 6 ans et enfin un épisode d’hématémèse. Elle présentait depuis 6 mois des douleurs para-ombilicales gauches intermittentes évoluant dans un contexte d'anorexie et d'amaigrissement non chiffré. L'examen abdominal retrouvait une volumineuse masse palpable, épigastrique, dure et mobile, de 15 cm de grand axe. Le bilan biologique objectivait une anémie hypochrome microcytaire à 8,7g/dl d’hémoglobine et une hyperleucocytose à 12000/mm3. La fibroscopie œsogastrique, réalisée chez notre patiente, trouvait une formation gastrique intraluminale faite de cheveux entrelacés posant le diagnostic de trichobezoar. Une extraction endoscopique est tentée, malheureusement sans succès. L'exérèse chirurgicale du trichobezoar est alors réalisée à travers une gastrotomie Les suites opératoires étaient simples et la fillette a été adressée en consultation psychiatrique.

**Observation nº2**

**Figure 1:**
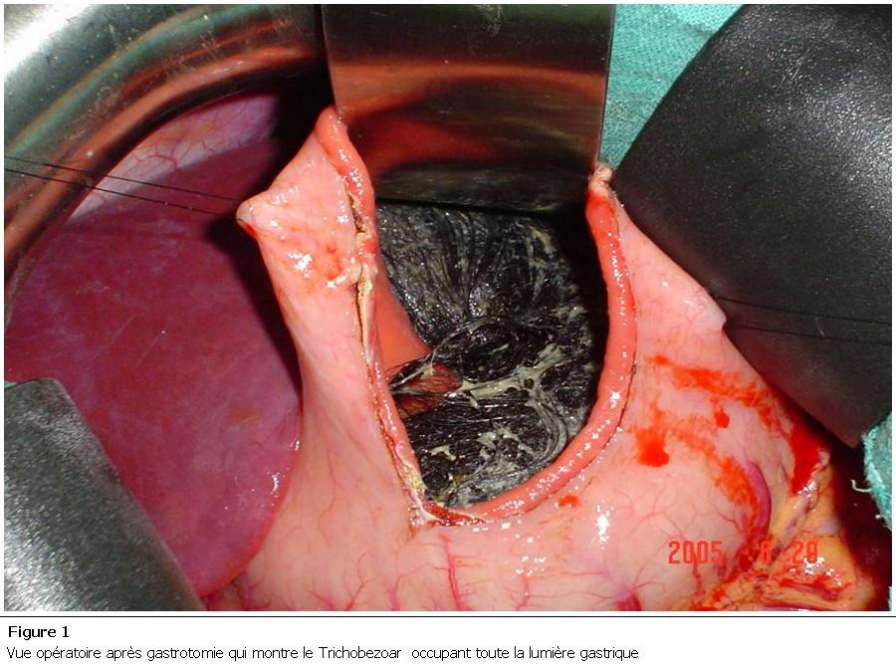
Vue opératoire après gastrotomie qui montre le trichobezoar occupant toute la lumière gastrique

Il s’agit d’une jeune de 18 ans, sans antécédents pathologiques notables, dont la symptomatologie remonte à 4 mois, par l’installation de douleurs abdominales diffuses atypiques prédominant au niveau de la région épigastrique, accompagnée de vomissements alimentaires post prandiales tardives, évoluant dans un contexte d’amaigrissement non chiffré. A l’examen on retrouve une énorme masse allant de l’hypochondre gauche à l’épigastre mesurant 15 cm, ferme, mobile et indolore. La fibroscopie oesogastroduodénale a révélé un Trichobezoar gastrique. L’échographie abdominale a objectivé une masse au dépend de l’estomac fléchissant les échos responsable d’un cône d’ombre postérieur. La patiente a été opérée. L’extraction du Trichobezoar a été réalisée à travers une large gastrotomie longitudinale (Figure 1 et 2). Les suites opératoires étaient simples. La patiente niant toute trichophagie a été adressé en consultation psychiatrique.

## Discussion

Le "Bézoard" issu du persan " Panzehr ", ou de l'arabe " Badzehr", signifie antidote ou antipoison [1]. Il désigne une affection rare, secondaire à l'accumulation inhabituelle, sous forme de masses solides ou de concrétions, de substances de diverses natures à l'intérieur du tube digestif et plus particulièrement au niveau de l'estomac. La nature de ces substances détermine le type du bézoard. Ainsi le trichobezoar qui représente 55% de tous les bézoards est fait de cheveux, poils ou fibres de tapis ou moquette de taille variable, entrelacés entre eux le plus souvent dans la lumière gastrique pouvant mouler celle-ci et avoir parfois des prolongements dans le duodénum, le jéjunum et même au-delà appelé“Syndrome de Rapunzel“ [2].

L'âge de survenue du Trichobezoar est dans 80% des cas inférieur à 30 ans avec un pic de fréquence entre 10 et 19 ans [3,4]. La prédominance féminine est nette (90% des cas). Le trichobezoar se voit souvent chez des patients émotionnellement perturbés ou déprimés et les prisonniers. Ces derniers avalent leurs cheveux (trichophagie) après les avoirs arrachés (trichotillomanie) [5]. Il peut survenir spontanément parfois chez les coiffeurs, les travailleurs de laine et les tisseurs de tapis [3]. Un certain nombre de facteurs favorisants ont été rapporté tels que des antécédents de chirurgie gastrique ou des troubles de la motilité gastrique [6]. Le trichobezoar peut rester asymptomatique pendant longtemps ou se manifester par une vague gène épigastrique (80%), des douleurs abdominales (70%), des nausées ou vomissements (65%), une asthénie avec amaigrissement (38%) ou des troubles du transit (33%) à type de diarrhée ou de constipation [3,7]. Plusieurs complications peuvent être le mode de révélation de cette pathologie [7]. Elle peut s’agir d’hémorragie digestive haute, due aux ulcérations pariétales, d'une occlusion mécanique, gastrique ou grélique [8], d’une perforation digestive responsable de péritonite ou abcès sous phrénique [3], d’une fistule digestive [2,3], de choléstase ou de pancréatite aiguë [7,9].

L'examen clinique retrouve, dans 87,7% des cas, une masse abdominale bien limitée, lisse, ferme, mobile à localisation épigastrique. Une alopécie ou une haleine fétide peuvent également être notées [3,7]. Le diagnostic repose sur la fibroscopie œso-gastro-duodénal qui reste l'examen de référence, il a un double intérêt, diagnostic tout d'abord permettant la visualisation d'un processus composé de cheveux enchevêtrés pathognomonique du trichobezoar, et thérapeutique par la suite réalisant l'extraction endoscopique de celui-ci [7].

L’échographie permet de poser le diagnostic dans 25% des cas, en visualisant une bande superficielle, hyperéchogène, curviligne avec cône d'ombre net postérieur [10,11]. Le transit œso-gastro-duodénal rarement utilisé objective une lacune intraluminale gastrique, à bords convexes, mobile, pouvant avoir des extensions dans le duodénum. Le Transit du grêle complète l’exploration de l’intestin à la recherche d’extension ou de fragments détachés.

La tomodensitométrie (TDM) visualise une masse intraluminale mobile, hétérogène avec souvent en son sein de petites collections de baryte provenant d'un transit antérieur [10]. A l’Imagerie par Résonance Magnétique (IRM), le trichobezoar a un aspect variable selon sa composition en air, eau, graisse et résidus alimentaires [9].

Plusieurs thérapeutiques ont été rapportées dans la littérature. Ainsi, en présence de trichobezoar de petite taille, certains auteurs proposent l'usage de boisson abondante associée à la prise d'accélérateurs du transit, en cas d’échec l'extraction endoscopique de celui-ci peut être tenté en s’aidant de rayon laser ou de mini explosion pour le fragmenté 12]. La lithotripsie extracorporelle a été proposé dans la littérature comme alternative [13], cependant ces sont souvent incomplet et exposent le patient à un grand risque d'occlusion intestinale sur fragment de trichobezoar.

Le traitement de choix reste la chirurgie conventionnelle ou cœlioscopique [14], permettant l'exploration de tout le tube digestif, l'extraction du trichobezoar gastrique à travers une gastrotomie ainsi que l'extraction d'éventuels prolongement (queue) ou fragments de celui-ci bloqués à distance de l’estomac à travers une ou plusieurs entérotomies [15].

Par ailleurs, une prise en charge psychiatrique des patients doit souvent être instaurée, avec rasage des cheveux de ceux ayant tendance à la trichophagie ou ayant déjà eu un Trichobezoar dans leurs antécédents.

## Conclusion

Le Trichobezoar reste une curiosité pathologique, du fait de sa nature et de sa rareté. Son diagnostic et son traitement simples ne doivent pourtant pas occulter l'éventuelle prise en charge psychiatrique des patients, qui reste difficilement applicable et acceptable dans notre contexte.

## Conflits d’intérêts

Les auteurs déclarent n’avoir aucuns conflits d’intérêts.

## Contribution des auteurs

**KIM** a rédigé l’article, HE a contribué à la prise des photos, **YB** a contribué à la recherche bibliographique, les autres auteurs ont contribué à la prise en charge thérapeutique de la malade et à la rédaction de ce document.

## Figures and Tables

**Figure 2:**
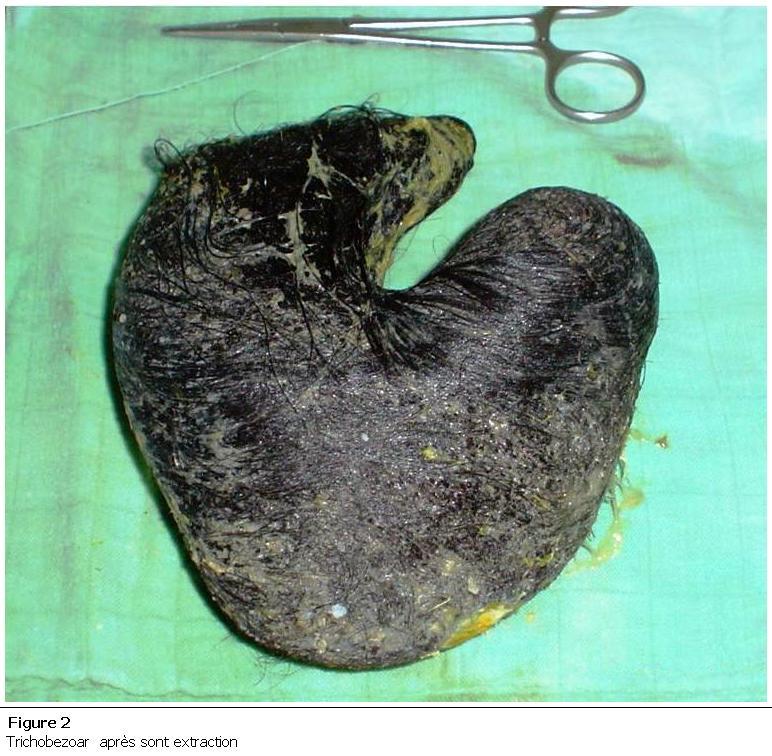
Trichobezoar après sont extraction
